# Adjuvant chemoradiotherapy in elderly patients with head and neck cancer: a monoinstitutional, two-to-one pair-matching analysis

**DOI:** 10.1007/s00066-021-01890-2

**Published:** 2022-01-17

**Authors:** Arnulf Mayer, Witali Wenzel, Daniel Wollschläger, Tilman Bostel, Maximilian Krüger, Christoph Matthias, Heinz Schmidberger

**Affiliations:** 1 Department of Radiation Oncology, University Medical Center, Johannes Gutenberg University, Mainz, Germany; 2 Institute of Medical Biostatistics, Epidemiology and Informatics (IMBEI), University Medical Center, Johannes Gutenberg University, Mainz, Germany; 3 Department of Oral and Maxillofacial Surgery – Plastic Surgery, University Medical Center, Johannes Gutenberg University, Mainz, Germany; 4 Department of Otorhinolaryngology, University Medical Center, Johannes Gutenberg University, Mainz, Germany

**Keywords:** Chemotherapy, Overall survival, Progression-free survival, Toxicity, Performance status

## Abstract

**Purpose:**

About one fifth of patients with head and neck cancer are aged 70 years and older at the time of diagnosis. In these patients, risk factors (R1 status or extracapsular extension of lymph node metastases, ECE) often lead to a need for combined chemoradiotherapy (CRT) in the postoperative setting. However, there is considerable concern about the toxicity of such therapy in this age group.

**Methods:**

Retrospective evaluation of the data of 53 patients ≥ 70 years of age who underwent surgery in our hospital between 1999 and 2015 for tumors of the oral cavity, the oropharynx, the hypopharynx, or the larynx, who subsequently received adjuvant radiation therapy. Two younger patients (< 70 years) were assigned to each of the elderly patients in a matching procedure based on anatomic sublocalization and tumor stage. The total cohort was comprised of 154 patients.

**Results:**

Univariate analyses revealed a statistically significant influence of many factors on overall survival (OS) and progression-free survival (PFS), including Karnofsky performance score (KPS), alcohol consumption, smoking, R status, ECE, chemotherapy, and discontinuation of RT. Younger patients had better OS and PFS compared to the elderly (*p* = 0.013 and 0.012, respectively). In a multivariate Cox regression, no independent influence of age on OS and PFS was found. Survival was primarily dependent on the addition of chemotherapy to radiotherapy (RT), application of the full course of RT, continued alcohol abuse, KPS, and the presence of ECE. Toxicity analysis showed a higher incidence of chronic renal failure but, generally, side effects for elderly patients were not substantially greater.

**Conclusion:**

Performance status and behavioral risk factors but not chronological age are crucial for the prognosis of patients who require adjuvant chemoradiation.

**Supplementary Information:**

The online version of this article (10.1007/s00066-021-01890-2) contains supplementary material, which is available to authorized users.

## Introduction

Two randomized clinical trials have demonstrated a survival benefit for the addition of chemotherapy to postoperative radiotherapy (RT) in cancers of the head and neck region [3, 6]. Both studies also showed a significant increase in overall acute therapy-associated toxicity, which may lead to treatment interruptions, a decrease in quality of life, and even therapy-associated deaths. The adverse effects associated with the addition of chemotherapy are also present in younger patients. However, they are expected to be much more severe in patients of a more advanced age. One of the two studies, by Cooper and coworkers [6], included only 7% elderly patients in the RT alone arm and 5% elderly patients in the arm treated with combined chemoradiotherapy (CRT). The other trial by Bernier et al. [3] did not include patients older than 70 years at all. For this reason, it is currently not proven that the addition of chemotherapy to postoperative radiation results in a survival benefit for elderly patients with head and neck cancers. Data from a large register in France show that the peak incidence of head and neck cancer is in the 55–59 years age group [18], while register data from Germany show the peak incidence in the 50–59 years age group [23]. However, in clinical practice, a substantial percentage of the patients who are presented at tumor conferences and discussed for adjuvant treatment after resection of squamous cell carcinomas of the oral cavity, the oropharynx, the hypopharynx, and the larynx fall into the category of patients aged 70 years or older (20.5% of all patients according to the Munich registry data [23]). There is little dispute about the feasibility of radiotherapy as such in elderly patients, and Kretschmer et al. have recently provided evidence for this being true in even the oldest old, i.e., ≥ 85 years of age [13].

Some clinicians advocate a more aggressive approach and routinely prescribe combined chemoradiation in the adjuvant setting for patients over 70 years of age (when indicated in case of R1 resection or extracapsular extension of lymph node metastases, ECE). They typically argue that the biological age of the patient is a critical factor. This opinion is based on the experience that chronological age, especially in light of a very different extent of cigarette smoking and alcohol consumption in the patient population, can deviate widely from the age-typical general condition of the corresponding chronological age. Other clinicians are less willing to take these risks in the absence of positive results from randomized trials for this specific patient population.

More data on the actual course of the disease and the extent of therapy-associated acute and late complications are needed for an optimal clinical decision in an individualized approach. In the present study, we have examined a cohort of 154 patients to determine to what extent the addition of chemotherapy to adjuvant radiotherapy has an impact on progression-free and overall survival rates. Additionally, we systematically explore the effect of simultaneous chemotherapy on the toxicity profile. For this purpose, we assigned two younger patients to each patient over 70 years of age in a matched-pair analysis.

## Patients and methods

### Study cohort

The final cohort analyzed retrospectively for this study from the clinical records consisted of 154 patients who had been treated at the Department of Radiation Oncology of the University Medical Center of the Johannes Gutenberg-University Mainz, Germany, between 1999 and 2015. The number of patients and the constitution of the cohort was the result of a selection process based on the total population of patients who had been treated at our clinic for tumors of the head and neck region during the period mentioned above. The selection process was based on the following criteria:

- squamous cell carcinoma of the oral cavity, oropharynx, hypopharynx, or larynx,
- curative-intent adjuvant radiotherapy or chemoradiotherapy indicated,
- Karnofsky performance score (KPS) > 50,
- no other synchronous malignant diseases.

A total of 874 patients with a tumor of the head and neck region were irradiated at our hospital between 1999 and 2015. Of these, 365 patients received primary radiotherapy or chemoradiotherapy, and 509 patients received adjuvant radiotherapy or chemoradiotherapy. Indication for chemotherapy was usually based on R1 resection status or extracapsular extension of lymph node metastases (ECE). However, the final decision was at the discretion of the treating physician. Of the patients receiving adjuvant therapy, 95 were excluded because their tumor was not located in the mentioned sublocalizations of the head and neck region. These cases were mostly primary tumors of the nasopharynx or salivary glands, nasal carcinomas, or squamous cell carcinomas of the ear. Furthermore, 29 patients had to be excluded as their tumors had histology other than squamous cell carcinoma, e.g., adenocarcinoma, lymphoma, plasmacytoma, or sarcoma. Another 32 patients were diagnosed with carcinoma of unknown primary (CUP) syndrome and were also not included in the study. Twenty-two patients had to be omitted because their radiotherapy was discontinued early, and curative intent was given up. Fourteen patients were excluded because they received non-cisplatin-based systemic therapy (e.g., cetuximab) simultaneously with radiation. No instances of re-irradiation were part of this study.

The final cohort for analysis thus consisted of 317 remaining patients, 53 (17%) of whom were 70 years or older (“elderly”), while 264 patients (83%) were aged under 70 years (“younger”). All 53 elderly patients were included in the final study cohort. Staging was done according to the 7th edition of the staging manual of the AJCC/UICC. These 53 elderly patients were matched according to UICC stage and subsite of the primary tumor with a group of young patients twice as large. A two-to-one pair-matching strategy, based on random numbers generated in Microsoft Excel (Microsoft, Redmond, WA, USA), was applied. Our initial evaluation showed some ambiguity regarding the definition and differentiation of oro- and hypopharyngeal carcinomas in the historical records. These two sublocalizations, although theoretically very different in biology, were combined into one group. The 2:1 matching was almost entirely feasible, except for four elderly patients with tumors of the oral cavity in stage I, who could only be assigned to five younger patients and also four elderly patients with laryngeal carcinomas in stage III. They could be assigned to only six younger patients exhibiting the same disease stage. In both cases, this was the maximum number of available cases among younger patients. The control group, therefore, included a total of 101 patients instead of 106 (154 patients in the entire study group, see above and Supplementary Table 1). Tumor and patient data of this cohort according to age groups are shown in Table 1 (Supplementary Table 2 presents the same information for the subset of 108 patients with an indication for chemotherapy). Therapy-associated side effects were recorded according to the Common Terminology Criteria for Adverse Events (CTCAE), version 4.0. A weighted toxicity score (WTS) was calculated for each category of side effect as the sum of the percentages of patients who developed this type and grade of toxicities, multiplied by a severity index corresponding to the grade level (e.g., WTS_Dermatitis_ = % of patients with grade 1 dermatitis * 1 + % of patients with grade 2 dermatitis * 2 + % of patients with grade 3 dermatitis * 3 + % of patients with grade 4 dermatitis * 4).

**Table 1:** Tumor and patient characteristics

Characteristic	*N*	< 70 years “young,” *N* = 101^a^	≥ 70 years “elderly,” *N* = 53^a^	*p*-value^b^	q‑value^c^
*Age*	154	(Mean, range)		–	–
	–	58.2 years	76.3 years	–	–
	–	(36.0 to 69.7)	(70.5 to 86.7)	–	–
*Karnofsky*	154	–	–	< 0.001	0.003
80+	–	85 (84%)	30 (57%)	–	–
≤ 70	–	16 (16%)	23 (43%)	–	–
*Gender*	154	–	–	0.57	0.73
Male	–	73 (72%)	36 (68%)	–	–
Female	–	28 (28%)	17 (32%)	–	–
*Presentation*	154	–	–	0.11	0.18
Primary	–	72 (71%)	29 (55%)	–	–
Recurrent	–	23 (23%)	20 (38%)	–	–
Multiple tumors	–	6 (5.9%)	4 (7.5%)	–	–
*Alcohol*	154	–	–	0.020	0.061
Never	–	58 (57%)	42 (79%)	–	–
Terminated	–	35 (35%)	10 (19%)	–	–
Current	–	8 (7.9%)	1 (1.9%)	–	–
*Smoker*	154	–	–	0.004	0.018
Never/nd	–	19 (19%)	20 (38%)	–	–
Terminated	–	62 (61%)	31 (58%)	–	–
Current	–	20 (20%)	2 (3.8%)	–	–
*T stage*	154	–	–	0.005	0.021
pT1	–	39 (39%)	11 (21%)	–	–
pT2	–	33 (33%)	12 (23%)	–	–
pT3	–	11 (11%)	15 (28%)	–	–
pT4a	–	18 (18%)	15 (28%)	–	–
*N stage (simplified)*	154	–	–	0.59	0.73
N0	–	37 (37%)	23 (43%)	–	–
N1	–	19 (19%)	7 (13%)	–	–
N2–3	–	45 (45%)	23 (43%)	–	–
*UICC*	154	–	–	0.97	> 0.99
I	–	15 (15%)	9 (17%)	–	–
II	–	6 (5.9%)	3 (5.7%)	–	–
III	–	22 (22%)	12 (23%)	–	–
IVa	–	58 (57%)	29 (55%)	–	–
*Localization*	154	–	–	0.97	> 0.99
Oral cavity	–	51 (50%)	27 (51%)	–	–
Oro-/hypopharynx	–	34 (34%)	17 (32%)	–	–
Larynx	–	16 (16%)	9 (17%)	–	–
*Resection*	154	–	–	> 0.99	> 0.99
R0	–	21 (21%)	11 (21%)	–	–
Close (< 5 mm)	–	55 (54%)	29 (55%)	–	–
R1	–	25 (25%)	13 (25%)	–	–
*Grading*	154	–	–	0.46	0.69
1	–	6 (5.9%)	2 (3.8%)	–	–
2	–	65 (64%)	30 (57%)	–	–
3	–	30 (30%)	21 (40%)	–	–
*Perineural spread*	154	–	–	0.048	0.10
Negative	–	97 (96%)	46 (87%)	–	–
Positive	–	4 (4.0%)	7 (13%)	–	–
*ECE*	154	–	–	0.038	0.094
Absent	–	92 (91%)	42 (79%)	–	–
Present	–	9 (8.9%)	11 (21%)	–	–
*Chemotherapy*	154	–	–	0.003	0.018
No ind.	–	32 (32%)	14 (26%)	–	–
Received	–	59 (58%)	22 (42%)	–	–
Ind., not received	–	10 (9.9%)	17 (32%)	–	–
*RT discontinuation*	154	–	–	0.064	0.12
Full course	–	98 (97%)	47 (89%)	–	–
Discontinued	–	3 (3.0%)	6 (11%)	–	–

*UICC* Union for International Cancer Control, *ECE* extracapsular extension of lymph node metastases, *RT* radiotherapy

^a^*n* (%)

^b^Pearson’s chi-squared test; Fisher’s exact test

^c^False discovery rate correction for multiple testing

### Treatment protocols

Until 2009, radiation was primarily applied as 3D conformal radiotherapy (57% of all patients). IMRT was used for most patients thereafter (42%). The majority of patients received a total dose of 64 Gy (64.2%). The remaining patients received total doses between 54 and 60 Gy, according to the individualized risk profile. Simultaneously integrated boost techniques with single doses exceeding 2.0 Gy were temporarily used in a minority of patients (8%). Chemotherapy was primarily based on cisplatin which was mainly applied in the first and the fifth week of adjuvant radiotherapy. Until 2004, patients received 20 mg/m^2^ body surface area daily for 3 days, corresponding to a total cumulative dose of 120 mg/m^2^. Beginning in 2005, patients received a daily dose of 20 mg/m^2^ KOF for 5 days in the first and fifth week of irradiation and, thus, a cumulative total dose of 200 mg/m^2^.

### Study endpoints

The primary endpoints of our analysis were overall survival (OS) and progression-free survival (PFS). OS was defined as the time from the first day of radiotherapy until the patient’s death from any cause. PFS was defined as the time from the first day of radiation until disease progression, either in the form of a locoregional recurrence or distant metastasis or both, or death from any cause. Survival curves were plotted using the Kaplan–Meier method, and differences between groups were studied using log-rank statistics. Additional univariate and multivariate survival analyses were done using Cox proportional hazard regression methods. As a sensitivity analysis, Cox regression was also carried out using a robust sandwich estimate for the covariance matrix to account for clustering by matching triplets. Differences between groups were considered significant if the *p*-value was less than 0.05. Exploratory data analysis and variable labeling were carried out in Jamovi 1.6.13.0 [20]. All calculations were carried out in R version 4.0.3 using the packages “survival” [21] and “survminer” [12]. Tables were formatted in “gtsummary” [17].

## Results

### Univariate survival analyses

The median follow-up time calculated according to the reverse Kaplan–Meier method of Schemper and Smith [16] was 79.9 months. At the time of data analysis, 79 of the 154 (51.3%) patients had died. Of these 79 patients, 32 (40.5%) died of a locoregional recurrence, 11 (13.9%) died with distant metastases (without a locoregional recurrence ever being detected), and a further 11 (13.9%) died of a second tumor. The remaining 25 (31.6%) patients either died of a disease not directly related to the malignant tumor or died of an unknown cause. The distribution of patients in the above categories for the two age groups (young patients versus older patients) is shown in Supplementary Fig. 1. One additional patient, who had not died at the time of data evaluation, also developed a recurrence. Thus, a total of 44 patients (29% of all patients) were diagnosed with a tumor recurrence, and the number of locoregional recurrences increased to a total of 33 (21% of all patients). The two age groups differed concerning a number of factors, as shown in Table 1.

The proportion of patients for whom chemotherapy was not considered indicated was roughly the same in both age groups. This proportion was 31.7% for the younger patients and 26.4% for the older ones. A difference was evident when comparing the proportions of patients with a chemotherapy indication based on the established risk factors (R1, extracapsular extension of lymph node metastases, ECE) who received it. The relative proportions of all patients in the respective groups were 85.5% for the younger patients, but only 56.4% for the older patients (corresponding to 14.5% and 43.6%, respectively, who had a chemotherapy indication but then did not receive it, see Supplementary Fig. 2).

For both OS and PFS, several influencing factors were examined in the univariate analyses. Fig. 1 shows Kaplan–Meier plots for the differences in OS of a selection of variables, most of them showing a strong influence on survival. These variables were age categories (3A), Karnofsky performance score (KPS, 3B), alcohol consumption (3C), smoking status (3D), resection status (3E), ECE (3F), chemotherapy (3G), and radiotherapy (RT) course (3H). Factors with a partially lower, but still statistically significant, influence on OS were perineural tumor invasion, T stage (T3 vs. T1), N‑stage (N1 vs. N0), UICC tumor stage (III vs. I), and initial presentation (multiple tumors vs. primary RT of a single tumor). These results are presented in Table 2. Practically identical results were obtained for progression-free survival. These can be found in Supplementary Table 3. Note that *p*-values in these tables are derived from the univariate Cox regressions, while the *p*-values given in Fig. 1 are from the log-rank tests. While based on the same data frame, numerical values vary slightly solely due to this methodological difference. The differences in survival between younger and elderly patients were pronounced. The younger patients had a median OS of 92.2 months compared to 35.0 months in the group of elderly patients (and 92.2 and 34.2 months for PFS of younger and elderly patients, respectively). OS at 1, 3, and 5 years was 87.1, 73.0, and 60.8%, respectively, for the younger patients. In elderly patients, these numbers were 81.1, 48.9, and 43.0%.

**Fig. 1:**
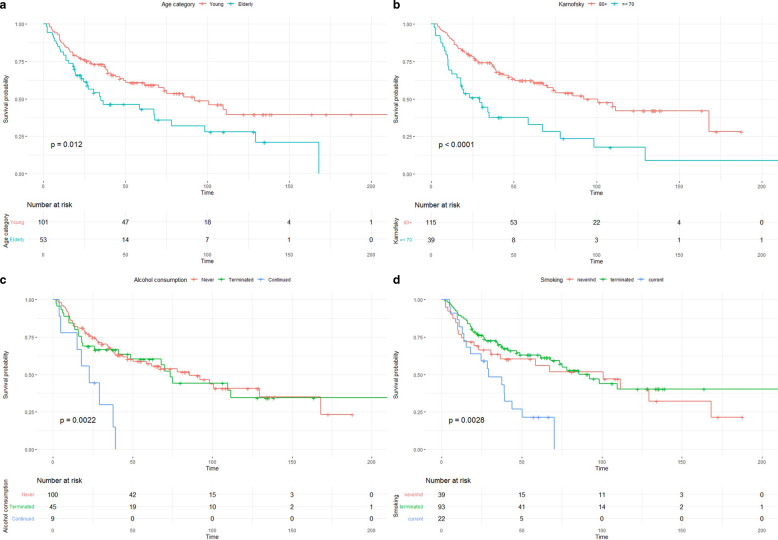
Kaplan–Meier plots for age categories (**a**), Karnofsky performance score (KPS, **b**), alcohol consumption (**c**), smoking (**d**), resection status (**e**), extracapsular extension of lymph node metastases (ECE, **f**), chemotherapy (**g**), and radiotherapy (RT) course (**h**)

**Fig. 1:**
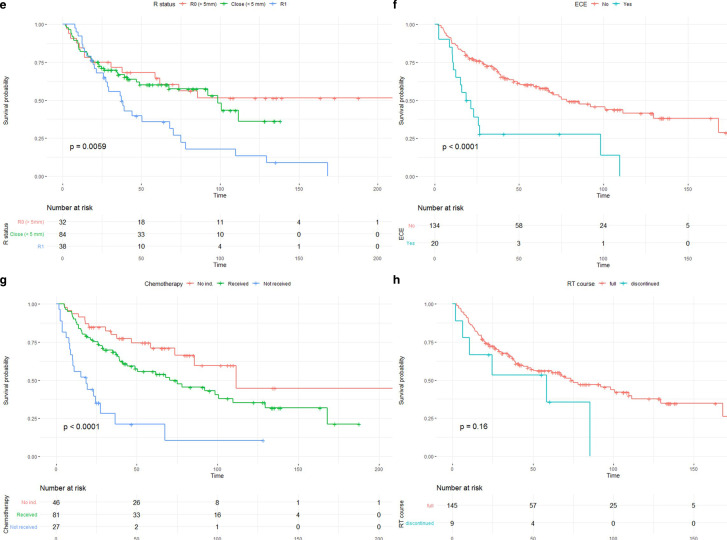
(Continued)

**Table 2:** Results of the univariate Cox regressions for overall survival

Characteristic	*N*	Event *N*	HR	95% CI	*p*-value	q‑value^a^
*Age group*	154	79	–	–	–	–
Young	–	–	–	–	–	–
Elderly	–	–	1.76	1.13, 2.77	0.013	0.041
*Karnofsky*	154	79	–	–	–	–
80+	–	–	–	–	–	–
≤ 70	–	–	2.46	1.55, 3.92	< 0.001	0.002
*Gender*	154	79	–	–	–	–
Male	–	–	–	–	–	–
Female	–	–	0.90	0.55, 1.47	0.67	0.75
*Presentation*	154	79	–	–	–	–
Primary	–	–	–	–	–	–
Recurrent	–	–	1.61	0.99, 2.63	0.057	0.12
Multiple tumors	–	–	2.62	1.22, 5.62	0.013	0.041
*Alcohol*	154	79	–	–	–	–
Never	–	–	–	–	–	–
Terminated	–	–	1.06	0.64, 1.74	0.83	0.86
Current	–	–	3.56	1.65, 7.65	0.001	0.008
*Smoker*	154	79	–	–	–	–
Never/nd	–	–	–	–	–	–
Terminated	–	–	0.82	0.48, 1.40	0.47	0.57
Current	–	–	2.17	1.12, 4.22	0.022	0.061
*T stage*	154	79	–	–	–	–
pT1	–	–	–	–	–	–
pT2	–	–	1.23	0.69, 2.19	0.49	0.57
pT3	–	–	2.36	1.23, 4.54	0.010	0.041
pT4a	–	–	1.54	0.82, 2.89	0.18	0.28
*N stage (simplified)*	154	79	–	–	–	–
N0	–	–	–	–	–	–
N1	–	–	1.82	1.02, 3.25	0.044	0.10
N2–3	–	–	0.99	0.60, 1.65	0.98	0.98
*UICC*	154	79	–	–	–	–
I	–	–	–	–	–	–
II	–	–	2.22	0.78, 6.28	0.13	0.25
III	–	–	2.71	1.26, 5.83	0.011	0.041
IVa	–	–	1.52	0.73, 3.15	0.26	0.39
*Localization*	154	79	–	–	–	–
Oral cavity	–	–	–	–	–	–
Oro-/hypopharynx	–	–	0.69	0.41, 1.14	0.14	0.25
Larynx	–	–	0.91	0.49, 1.68	0.76	0.82
*Resection*	154	79	–	–	–	–
R0	–	–	–	–	–	–
Close (< 5 mm)	–	–	1.32	0.70, 2.47	0.39	0.52
R1	–	–	2.47	1.29, 4.72	0.006	0.034
*Grading*	154	79	–	–	–	–
1	–	–	–	–	–	–
2	–	–	1.70	0.53, 5.45	0.37	0.52
3	–	–	1.57	0.48, 5.21	0.46	0.57
*Perineural*	154	79	–	–	–	–
Negative	–	–	–	–	–	–
Positive	–	–	2.49	1.13, 5.50	0.024	0.062
*ECE*	154	79	–	–	–	–
Absent	–	–	–	–	–	–
Present	–	–	2.90	1.66, 5.08	< 0.001	0.002
*Chemotherapy*	154	79	–	–	–	–
No ind.	–	–	–	–	–	–
Received	–	–	1.76	0.98, 3.17	0.059	0.12
Ind., not received	–	–	5.52	2.78, 11.0	< 0.001	< 0.001
*RT course*	154	79	–	–	–	–
Full course	–	–	–	–	–	–
Discontinued	–	–	1.81	0.79, 4.19	0.16	0.27

*HR* hazard ratio, *CI* confidence interval, *UICC* Union for International Cancer Control, *ECE* extracapsular extension of lymph node metastases, *RT* radiotherapy

^a^False discovery rate correction for multiple testing

### Multivariate survival analysis

In the next step, we performed a multivariate Cox regression which included the two age groups, KPS, alcohol consumption, resection status, ECE, chemotherapy application, and discontinuation of the RT course. Smoking status was left out because of incomplete data and substantial overlap with alcohol consumption. Results show that not receiving chemotherapy despite having an indication for it was associated with a more than quadrupled hazard rate (HR 4.19, *p* < 0.001). In multivariate analysis, continued alcohol consumption had an HR only slightly lower for OS (HR 4.02, *p* < 0.001). Discontinuation of RT had an effect of comparable magnitude (HR 3.56, *p* = 0.007). KPS and ECE also retained an independent influence on OS, while the resection status (R1 vs. R0) missed the level of statistical significance (see Table 3). Results of the same analysis for PFS were practically identical except for the resection status (R1 vs. R0), which retained its statistically independent influence on PFS (see Supplementary Table 4). Cox regressions were also calculated using the triplet (one elderly, two young patients) identifier as a cluster variable. Numeric results were nearly identical (data not shown). Furthermore, univariate and multivariate survival analyses were also carried out in the subset of 108 patients with an indication for chemotherapy. Conclusions from the data remain the same (Supplementary Table 5).

**Table 3:** Results of the multivariate Cox regression for overall survival

Characteristic	HR	95% CI	*p*-value
*Age groups*			
Young	–	–	–
Elderly	1.00	0.58, 1.71	> 0.9
*Karnofsky*			
80+	–	–	–
≤ 70	2.36	1.40, 3.95	0.001
*Alcohol*			
Never	–	–	–
Terminated	1.16	0.68, 1.98	0.6
Current	4.02	1.81, 8.96	< 0.001
*Resection*			
R0	–	–	–
Close (< 5 mm)	1.07	0.55, 2.08	0.8
R1	1.93	0.93, 4.01	0.077
*ECE*			
Absent	–	–	–
Present	2.07	1.14, 3.75	0.017
*Chemotherapy*			
No ind.	–	–	–
Received	1.61	0.80, 3.21	0.2
Ind., not received	4.19	1.90, 9.26	< 0.001
*RT discontinuation*			
Full course	–	–	–
Discontinued	3.56	1.40, 9.02	0.007

*HR* hazard ratio, *CI* confidence interval, *ECE* extracapsular extension of lymph node metastases, *RT* radiotherapy

### Toxicity analysis

**Fig. 2:**
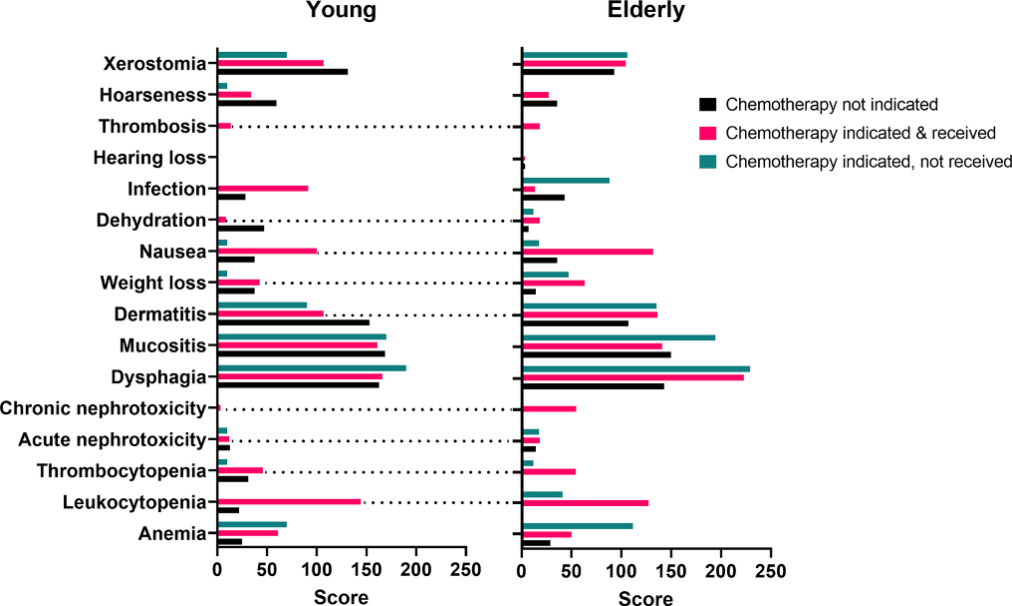
Comparative toxicity analyses in the groups of young and elderly patients. *Dashed horizontal lines* indicate toxicity categories in which the elderly patients receiving chemotherapy had the highest scores compared with both the younger patients and elderly patients who did not receive chemotherapy

The toxicity analysis is summarized in Fig. 2 and Supplementary Table 6. We compared adverse effects in elderly patients treated with chemotherapy with (i) younger patients who also received chemotherapy and (ii) older patients who did not receive chemotherapy. We analyzed acute thrombocytopenia, acute nephrotoxicity, chronic nephrotoxicity, dermatitis, weight loss, nausea, dehydration, and thrombosis. Chronic nephrotoxicity in elderly patients stands out with a score of 54.6. The corresponding score for younger patients is only 3.4. This was the only category for which a significant difference between younger and elderly patients was found (*p* = 0.004). Overall, it was evident that the toxicity profile of young and elderly patients did not differ substantially. The sum of the WTS for young and elderly patients treated with chemotherapy was 1097 vs. 1183, respectively. Hence, side effects were, in general, slightly less pronounced in young patients compared to the elderly; however, the difference was marginal.

## Discussion

The indication for adjuvant chemoradiation for tumors of the head and neck region is usually based on the presence of an extracapsular extension of lymph node metastases (ECE) and/or an R1 status. This approach is based on evidence from two randomized trials [2, 3, 8] (see the Introduction).

Several analyses of large cancer databases regarding the benefit of chemoradiation in the elderly in the presence of the classical risk factors (R1, ECE) have yielded heterogeneous results. Yoshida and colleagues [25] evaluated the data of 1199 patients aged 70 and above from the National Cancer Database (NCDB). After a median follow-up of 42.6 months, multivariate analysis showed improved overall survival with postoperative simultaneous chemoradiotherapy compared to radiotherapy alone in stage N2 to N3 patients. ECE had no independent influence on survival [25]. Woody and co-workers from Cleveland [24] investigated CRT compared to RT alone in patients with tumors of the oral cavity, oropharynx, larynx, and hypopharynx who had been treated with primary surgery and adjuvant radiotherapy between 2004 and 2012. In particular, they examined the results of older patients (over 70 years of age) whose histopathological findings had shown ECE or positive resection margins. 1187 patients with a median follow-up time of 30.6 months could be included in the analysis. Combined adjuvant CRT was correlated with a significant benefit in overall survival compared to RT alone (HR 0.74, *p* = 0.04). However, after propensity score matching, the addition of chemotherapy no longer resulted in a clear survival advantage.

A similar study was performed by Giacalone and collaborators [9]. From 1998 to 2011, these authors identified a total of 1686 elderly patients with positive surgical resection margins and/or ECE who had received adjuvant CRT (491 patients, 29%) or adjuvant RT alone (1195 patients, 71%). While univariate analysis also showed a significant survival benefit in favor of patients treated with combined adjuvant CRT, this difference was not maintained in a multivariate exploration. It was also eliminated after propensity score matching.

The indication for adjuvant CRT may also be based on other factors in an individualized fashion. Trifiletti et al. [22] evaluated data from the NCDB from 2004 to 2012. They identified 10870 patients with head and neck cancer who had received adjuvant RT or CRT without R1 status or ECE. A statistically significant advantage in terms of overall survival for combined CRT compared to RT alone was found. However, older patients (65 years and above) received combined CRT less often and were treated more frequently with RT alone. Conversely, Chen and colleagues from Stanford specifically addressed the question of whether adjuvant CRT offers a better outcome than adjuvant radiotherapy alone in patients without ECE and without a local R1 status [5]. They divided this analysis into two age groups. Patients aged 70 years and above were considered separately. For the latter group of patients, there was no overall survival benefit. Notably, for patients with HPV-positive oropharyngeal carcinoma, there was even a survival detriment for patients who had received adjuvant CRT.

We have found only two recent retrospective clinical studies that have also directly dealt with the influence of age on the indication for adjuvant CRT. Airoldi et al. [1] examined 40 patients aged 70 to 78 years who had received adjuvant CRT after radical surgery for tumors of the oral cavity, oropharynx, hypopharynx, and larynx. These authors used carboplatin instead of cisplatin. Their data on OS (3-year OS, 64%) were compared with the results of earlier work from the same group. They found that the addition of chemotherapy was beneficial in older patients. Their findings were comparable with younger patients who also received combined postoperative CRT. Haehl and colleagues [10] recently published their analysis of 246 patients aged 65 years or over, of whom about one third (*n* = 80) received adjuvant RT or CRT. For adjuvant patients between 65 and 74 years of age, they found only a trend towards better OS for CRT compared with RT alone. Interestingly, for patients aged 75 years and older, data showed an inferior OS for patients treated adjuvantly with CRT compared with those treated with RT alone. This trend persisted in the multivariate analysis (*p* = 0.072). Additionally, Nguyen et al. [14] evaluated the feasibility of chemoradiotherapy in a smaller cohort of elderly patients (*n* = 26). Contrary to our study, however, patients were treated with primary, definitive CRT. Nevertheless, the conclusions were similar.

A large meta-analysis of chemotherapy for tumors of the head and neck region (MACH-NC), which evaluated individual patient data of 16,485 patients from 87 randomized trials [15], showed no or only a small benefit for chemotherapy in patients aged 71 years and older (the 95% confidence interval in this subgroup crossed 1). Moreover, the benefit of the addition of chemotherapy in the adjuvant setting in this analysis is even questionable for younger patients.

Different effects may generally be responsible for the absence of a favorable impact of chemotherapy in older patients. For example, patients of advanced age die more frequently from causes other than their tumor disease, so that the potential benefit of the therapy may no longer become manifest. However, it must be borne in mind that older patients—although their absolute number was large—were underrepresented in the randomized trials analyzed in the MACH-NC meta-analysis (only 8% of the analyzed population, [15]. Their actual proportion is larger, ~ 24% of all head and neck cancer patients according to one source [7]).

In clinical practice, the addition of chemotherapy to adjuvant radiotherapy is regularly discussed due to the known biological aggressiveness of tumors of the head and neck region, at least as far as HPV-negative subtypes are concerned. On the one hand, in the case of tumor relapse, the disease is usually not curable, and, on the other hand, therapeutic alternatives are not available for these patients. The combination of cetuximab with radiation is not established in the postoperative setting outside of clinical trials. However, even in primary CRT, cetuximab is not a confirmed substitute for cisplatin for older patients [4].

Concerning the oncological benefit of chemotherapy in older patients with a given indication, our data provide a clear signal. In multivariate analysis, the administration of chemotherapy with a hazard ratio of 4.19 (*p* < 0.001) was the most potent positive influencing factor of OS amenable to modification.

Although the comparison of the toxicity profiles of the older and younger patients did not show any alarming differences, the severity of side effects was, however, slightly increased overall in the older patients. No therapy-associated deaths occurred. OS and PFS were very similar. These outcomes are likely the result of a careful selection process. It is noteworthy that our multivariate analysis clearly showed that performance status has an evident influence on OS and PFS, but surprisingly, belonging to one of the two age groups did not.

In summary, our data underline the appropriateness of the view that rigid cutoffs for adjuvant chemoradiation in head and neck cancer based on chronological age seem to be inferior to patient selection according to medical criteria. Carefully selected elderly patients derive a clear benefit from adjuvant chemoradiation. Furthermore, patients should receive professional counseling regarding alcohol abuse and should be referred to a smoking cessation assistance program [11]. The use of geriatric assessments [19] could also be helpful. All results should be interpreted with caution given the limitations of our study which include its retrospective nature, the small patient number, and the long period of data collection during which substantial improvements in the quality of radiotherapy planning and application took place.

## Supplementary Information

Supplementary Table 1. Details of the 2:1 matching process.
Supplementary Table 2. Patient and tumor characteristics according to the application of chemotherapy when indicated
Supplementary Table 3. Results of the univariate Cox regressions for progression-free survival
Supplementary Table 4. Results of the multivariate Cox regression for progression-free survival
Supplementary Table 5. Chemotherapy dichotomized into indicated & received (reference category) vs. indicated, but received
Supplementary Table 6. Distribution of treatment side effects according to the two age groups.

Supplementary Fig. 1. Distributions of survival status and causes of death for the two age groups

Supplementary Fig. 2. Indications for chemotherapy and the use of chemotherapy in the two age groups
