# Supplementary material for: Adjuvant chemoradiotherapy in elderly patients with head and neck cancer: a monoinstitutional, two-to-one pair-matching analysis
Source: Strahlenther Onkol. 2022 Jan 17;198(2):159–70. doi: 10.1007/s00066-021-01890-2 (PMC8789714; doi:10.1007/s00066-021-01890-2)
Supplement: Supplementary file 1 — Supplementary Table 1. Details of the 2:1 matching process. Supplementary Table 2. Patient and tumor characteristics according to the application of chemotherapy when indicated Supplementary Table 3. Results of the univariate Cox regressions for progression-free survival Supplementary Table 4. Results of the multivariate Cox regression for progression-free survival Supplementary Table 5. Chemotherapy dichotomized into indicated & received (reference category) vs. indicated, but received Supplementary Table 6. Distribution of treatment side effects according to the two age groups. [file 66_2021_1890_MOESM1_ESM.docx]

**Supplementary Tables**

**Supplementary Table 1. Details of the 2:1 matching process.**

| **UICC:** | | Elderly patients | Selected Young patients | All young patients |
| --- | --- | --- | --- | --- |
| 1. Oral: | I  II  III  IV a  IVb+c | 4  2  5  16  0 | 5*  4  10  32  0 | 5  16  19  51  5 |
| 1. Oro-   /hypo-  pharynx: | I  II  III  IV a  IVb+c | 4  0  3  10  0 | 8  0  6  20  0 | 12  13  34  65  5 |
| 1. Larynx: | I  II  III  IV a  IVb+c | 1  1  4  3  0 | 2  2  6*  6  0 | 5  2  6  26  0 |
| Total: | | 53 | 101 | 264 |

**Supplementary Table 2. Patient and tumor characteristics according to the application of chemotherapy when indicated.**

| **Characteristic** | **N** | **Chemo indicated & given N = 81 (75%)** | **Chemo indicated, but not given N = 27 (25%)** | | **p-value** | **q-value** |
| --- | --- | --- | --- | --- | --- | --- |
| Age | 108 | 62 (55, 71) | 72 (64, 79) | |  | 0.002 |
| Karnofsky | 108 |  |  | | 0.002 | 0.010 |
| 80+ |  | 68 (84%) | 14 (52%) | |  |  |
| =< 70 |  | 13 (16%) | 13 (48%) | |  |  |
| Gender | 108 |  |  | | >0.99 | >0.99 |
| male |  | 61 (75%) | 21 (78%) | |  |  |
| female |  | 20 (25%) | 6 (22%) | |  |  |
| Presentation | 108 |  |  | | 0.29 | 0.58 |
| primary |  | 55 (68%) | 14 (52%) | |  |  |
| recurrent |  | 21 (26%) | 11 (41%) | |  |  |
| multiple tumors |  | 5 (6.2%) | 2 (7.4%) | |  |  |
| Alcohol | 108 |  |  | | 0.94 | >0.99 |
| never |  | 54 (67%) | 17 (63%) | |  |  |
| terminated |  | 22 (27%) | 8 (30%) | |  |  |
| current |  | 5 (6.2%) | 2 (7.4%) | |  |  |
| Smoker | 108 |  |  | | 0.55 | 0.74 |
| never/nd |  | 19 (23%) | 9 (33%) | |  |  |
| terminated |  | 48 (59%) | 15 (56%) | |  |  |
| current |  | 14 (17%) | 3 (11%) | |  |  |
| T stage | 108 |  |  | | 0.37 | 0.59 |
| pT1 |  | 20 (25%) | 9 (33%) | |  |  |
| pT2 |  | 30 (37%) | 7 (26%) | |  |  |
| pT3 |  | 16 (20%) | 3 (11%) | |  |  |
| pT4a |  | 15 (19%) | 8 (30%) | |  |  |
| N stage simplified | 108 |  |  | | 0.60 | 0.74 |
| N0 |  | 20 (25%) | 9 (33%) | |  |  |
| N1 |  | 12 (15%) | 4 (15%) | |  |  |
| N2-3 |  | 49 (60%) | 14 (52%) | |  |  |
| UICC | 108 |  |  | | 0.37 | 0.59 |
| I |  | 5 (6.2%) | 4 (15%) | |  |  |
| II |  | 3 (3.7%) | 2 (7.4%) | |  |  |
| III |  | 17 (21%) | 4 (15%) | |  |  |
| IVa |  | 56 (69%) | 17 (63%) | |  |  |
| Localization | 108 |  | |  | 0.23 | 0.53 |
| OralCavity |  | 37 (46%) | | 17 (63%) |  |  |
| Oro-/Hypopharynx |  | 29 (36%) | | 8 (30%) |  |  |
| Larynx |  | 15 (19%) | | 2 (7.4%) |  |  |
| Resection | 108 |  |  | | 0.42 | 0.62 |
| R0 |  | 16 (20%) | 3 (11%) | |  |  |
| close (< 5 mm) |  | 36 (44%) | 16 (59%) | |  |  |
| R1 |  | 29 (36%) | 8 (30%) | |  |  |
| Grading | 108 |  |  | | >0.99 | >0.99 |
| 1 |  | 2 (2.5%) | 0 (0%) | |  |  |
| 2 |  | 50 (62%) | 17 (63%) | |  |  |
| 3 |  | 29 (36%) | 10 (37%) | |  |  |
| Perineural spread | 108 |  |  | | 0.12 | 0.37 |
| negative |  | 76 (94%) | 22 (81%) | |  |  |
| positive |  | 5 (6.2%) | 5 (19%) | |  |  |
| ECE | 108 |  |  | | 0.15 | 0.41 |
| absent |  | 69 (85%) | 19 (70%) | |  |  |
| present |  | 12 (15%) | 8 (30%) | |  |  |
| RTdiscontinuation | 108 |  |  | | 0.10 | 0.37 |
| full course |  | 79 (98%) | 24 (89%) | |  |  |
| discontinued |  | 2 (2.5%) | 3 (11%) | |  |  |
| ^1^ Statistics presented: median (IQR); n (%) | | | | | | |
| ^2^ Statistical tests performed: Wilcoxon rank-sum test; Fisher's exact test; chi-square test of independence | | | | | | |
| ^3^ False discovery rate correction for multiple testing | | | | | | |

**Supplementary Table 3**. Results of the univariate Cox regressions for progression-free survival.

| Characteristic | N | Event N | HR^1^ | 95% CI^1^ | p-value | q-value^2^ |
| --- | --- | --- | --- | --- | --- | --- |
| **AgeGroups** | 154 | 80 |  |  |  |  |
| *young* |  |  | — | — |  |  |
| *elderly* |  |  | 1.77 | 1.14, 2.77 | 0.012 | 0.038 |
| **Karnofsky** | 154 | 80 |  |  |  |  |
| *80+* |  |  | — | — |  |  |
| *=< 70* |  |  | 2.61 | 1.65, 4.13 | <0.001 | <0.001 |
| **Gender** | 154 | 80 |  |  |  |  |
| *male* |  |  | — | — |  |  |
| *female* |  |  | 0.89 | 0.54, 1.45 | 0.64 | 0.71 |
| **Presentation** | 154 | 80 |  |  |  |  |
| *primary* |  |  | — | — |  |  |
| *recurrent* |  |  | 1.67 | 1.03, 2.71 | 0.038 | 0.092 |
| *multiple tumors* |  |  | 2.64 | 1.24, 5.65 | 0.012 | 0.038 |
| **Alcohol** | 154 | 80 |  |  |  |  |
| *never* |  |  | — | — |  |  |
| *terminated* |  |  | 1.02 | 0.62, 1.67 | 0.94 | 0.94 |
| *current* |  |  | 3.74 | 1.74, 8.02 | <0.001 | 0.005 |
| **Smoker** | 154 | 80 |  |  |  |  |
| *never/nd* |  |  | — | — |  |  |
| *terminated* |  |  | 0.75 | 0.45, 1.27 | 0.29 | 0.40 |
| *current* |  |  | 1.99 | 1.03, 3.84 | 0.039 | 0.092 |
| **Tstage** | 154 | 80 |  |  |  |  |
| *pT1* |  |  | — | — |  |  |
| *pT2* |  |  | 1.24 | 0.69, 2.21 | 0.47 | 0.55 |
| *pT3* |  |  | 2.31 | 1.20, 4.42 | 0.012 | 0.038 |
| *pT4a* |  |  | 1.61 | 0.87, 2.99 | 0.13 | 0.22 |
| **N stage (simplified)** | 154 | 80 |  |  |  |  |
| *N0* |  |  | — | — |  |  |
| *N1* |  |  | 1.68 | 0.94, 2.99 | 0.079 | 0.16 |
| *N2-3* |  |  | 0.93 | 0.56, 1.55 | 0.79 | 0.82 |
| **UICC** | 154 | 80 |  |  |  |  |
| *I* |  |  | — | — |  |  |
| *II* |  |  | 2.35 | 0.83, 6.67 | 0.11 | 0.20 |
| *III* |  |  | 2.68 | 1.25, 5.75 | 0.012 | 0.038 |
| *IVa* |  |  | 1.57 | 0.76, 3.24 | 0.23 | 0.34 |
| **Localization** | 154 | 80 |  |  |  |  |
| *OralCavity* |  |  | — | — |  |  |
| *Oro-/Hypopharynx* |  |  | 0.67 | 0.40, 1.10 | 0.12 | 0.20 |
| *Larynx* |  |  | 0.89 | 0.48, 1.64 | 0.71 | 0.77 |
| **Resection** | 154 | 80 |  |  |  |  |
| *R0* |  |  | — | — |  |  |
| *close (< 5 mm)* |  |  | 1.28 | 0.68, 2.40 | 0.44 | 0.55 |
| *R1* |  |  | 2.67 | 1.40, 5.09 | 0.003 | 0.016 |
| **Grading** | 154 | 80 |  |  |  |  |
| *1* |  |  | — | — |  |  |
| *2* |  |  | 1.71 | 0.53, 5.49 | 0.37 | 0.49 |
| *3* |  |  | 1.55 | 0.47, 5.14 | 0.47 | 0.55 |
| **Perineural** | 154 | 80 |  |  |  |  |
| *negative* |  |  | — | — |  |  |
| *positive* |  |  | 2.31 | 1.05, 5.08 | 0.038 | 0.092 |
| **ECE** | 154 | 80 |  |  |  |  |
| *absent* |  |  | — | — |  |  |
| *present* |  |  | 2.65 | 1.52, 4.62 | <0.001 | 0.005 |
| **Chemotherapy** | 154 | 80 |  |  |  |  |
| *No ind.* |  |  | — | — |  |  |
| *Received* |  |  | 1.77 | 0.98, 3.19 | 0.057 | 0.12 |
| *Ind., not received* |  |  | 5.36 | 2.72, 10.6 | <0.001 | <0.001 |
| **RTdiscont** | 154 | 80 |  |  |  |  |
| *full course* |  |  | — | — |  |  |
| *discontinued* |  |  | 1.66 | 0.72, 3.84 | 0.23 | 0.34 |
| ^1^HR = Hazard Ratio, CI = Confidence Interval | | | | | | |
| ^2^False discovery rate correction for multiple testing | | | | | | |

**Supplementary Table 4**. Results of the multivariate Cox regression for progression-free survival.

| Characteristic | HR^1^ | 95% CI^1^ | p-value |
| --- | --- | --- | --- |
| **AgeGroups** |  |  |  |
| *young* | — | — |  |
| *elderly* | 0.91 | 0.54, 1.55 | 0.7 |
| **Karnofsky** |  |  |  |
| *80+* | — | — |  |
| *=< 70* | 2.95 | 1.76, 4.94 | <0.001 |
| **Alcohol** |  |  |  |
| *never* | — | — |  |
| *terminated* | 1.12 | 0.65, 1.91 | 0.7 |
| *current* | 5.14 | 2.32, 11.4 | <0.001 |
| **Resection** |  |  |  |
| *R0* | — | — |  |
| *close (< 5 mm)* | 1.08 | 0.55, 2.10 | 0.8 |
| *R1* | 2.37 | 1.14, 4.96 | 0.021 |
| **ECE** |  |  |  |
| *absent* | — | — |  |
| *present* | 1.91 | 1.06, 3.45 | 0.030 |
| **Chemotherapy** |  |  |  |
| *No ind.* | — | — |  |
| *Received* | 1.54 | 0.76, 3.09 | 0.2 |
| *Ind., not received* | 3.88 | 1.77, 8.51 | <0.001 |
| **RTdiscont** |  |  |  |
| *full course* | — | — |  |
| *discontinued* | 3.41 | 1.34, 8.67 | 0.010 |
| ^1^HR = Hazard Ratio, CI = Confidence Interval | | | |

**Supplementary Table 5.** Chemotherapy dichotomized into indicated & received (reference category) vs. indicated, but received.

|  |  | **all** | **HR (univariable)** | **HR (multivariable)** |
| --- | --- | --- | --- | --- |
| AgeGroups | young | 69 (100.0) | - | - |
|  | elderly | 39 (100.0) | 1.84 (1.12-3.03, p=0.016) | 0.85 (0.46-1.59, p=0.613) |
| Karnofsky | 80+ | 82 (100.0) | - | - |
|  | =< 70 | 26 (100.0) | 2.65 (1.56-4.50, p | 2.42 (1.28-4.55, p=0.006) |
| Alcohol | never | 71 (100.0) | - | - |
|  | terminated | 30 (100.0) | 1.04 (0.59-1.81, p=0.903) | 1.13 (0.61-2.09, p=0.706) |
|  | current | 7 (100.0) | 2.83 (1.18-6.80, p=0.020) | 3.25 (1.30-8.12, p=0.011) |
| Resection | R0 | 19 (100.0) | - | - |
|  | close (< 5 mm) | 52 (100.0) | 2.27 (0.97-5.27, p=0.057) | 1.30 (0.53-3.18, p=0.568) |
|  | R1 | 37 (100.0) | 2.84 (1.22-6.59, p=0.015) | 2.26 (0.94-5.44, p=0.068) |
| ECE | absent | 88 (100.0) | - | - |
|  | present | 20 (100.0) | 2.35 (1.32-4.19, p=0.004) | 2.14 (1.17-3.91, p=0.013) |
| RT discontinuation | full course | 103 (100.0) | - | - |
|  | discontinued | 5 (100.0) | 4.02 (1.42-11.39, p=0.009) | 6.99 (2.10-23.26, p=0.002) |
| Chemotherapy | indicated, received | 81 (100.0) | - | - |
|  | indicated, not received | 27 (100.0) | 3.13 (1.80-5.45, p | 2.58 (1.37-4.86, p=0.003) |

**Supplementary Table 6**. Distribution of treatment side effects according to the two age groups.

|  | **Grade** | **Younger vs. elderly patients** | | | | **Chi-square p** |
| --- | --- | --- | --- | --- | --- | --- |
|  |  | **< 70 years „young“** | | **>= 70 years. "elderly"** | |  |
|  |  | **Count** | **%** | **Count** | **%** |  |
| Anemia | 0 | 64 | 63.4% | 29 | 54.7% | n.s. |
|  | 1 | 25 | 24.8% | 15 | 28.3% |  |
|  | 2 | 10 | 9.9% | 8 | 15.1% |  |
|  | 3 | 2 | 2.0% | 1 | 1.9% |  |
| Leukopenia | 0 | 55 | 54.5% | 36 | 67.9% | n.s. |
|  | 1 | 11 | 10.9% | 3 | 5.7% |  |
|  | 2 | 25 | 24.8% | 10 | 18.9% |  |
|  | 3 | 9 | 8.9% | 4 | 7.5% |  |
|  | 4 | 1 | 1.0% | 0 | 0.0% |  |
| Thrombocytopenia | 0 | 80 | 79.2% | 42 | 79.2% | n.s. |
|  | 1 | 14 | 13.9% | 9 | 17.0% |  |
|  | 2 | 6 | 5.9% | 1 | 1.9% |  |
|  | 3 | 1 | 1.0% | 1 | 1.9% |  |
| Acute nephrotoxicity | 0 | 85 | 84.2% | 42 | 79.2% | n.s. |
|  | 1 | 14 | 13.9% | 10 | 18.9% |  |
|  | 2 | 1 | 1.0% | 1 | 1.9% |  |
|  | 3 | 1 | 1.0% | 0 | 0.0% |  |
| Chronic neprotoxicity | 0 | 99 | 98.0% | 45 | 84.9% | 0.004 |
|  | 1 | 2 | 2.0% | 4 | 7.5% |  |
|  | 2 | 0 | 0.0% | 4 | 7.5% |  |
| Dysphagia | 0 | 14 | 13.9% | 5 | 9.4% | n.s.  (0.064) |
|  | 1 | 22 | 21.8% | 5 | 9.4% |  |
|  | 2 | 48 | 47.5% | 26 | 49.1% |  |
|  | 3 | 17 | 16.8% | 17 | 32.1% |  |
| Mucositis | 0 | 10 | 9.9% | 11 | 20.8% | n.s. |
|  | 1 | 34 | 33.7% | 11 | 20.8% |  |
|  | 2 | 39 | 38.6% | 19 | 35.8% |  |
|  | 3 | 18 | 17.8% | 12 | 22.6% |  |
| Dermatitis | 0 | 15 | 14.9% | 7 | 13.2% | n.s. |
|  | 1 | 52 | 51.5% | 25 | 47.2% |  |
|  | 2 | 33 | 32.7% | 20 | 37.7% |  |
|  | 3 | 1 | 1.0% | 1 | 1.9% |  |
| Weight loss | 0 | 72 | 71.3% | 32 | 60.4% | n.s. |
|  | 1 | 21 | 20.8% | 18 | 34.0% |  |
|  | 2 | 7 | 6.9% | 3 | 5.7% |  |
|  | 3 | 1 | 1.0% | 0 | 0.0% |  |
| Nausea | 0 | 49 | 48.5% | 29 | 54.7% | n.s. |
|  | 1 | 32 | 31.7% | 11 | 20.8% |  |
|  | 2 | 20 | 19.8% | 13 | 24.5% |  |
| Dehydration | 0 | 91 | 90.1% | 49 | 92.5% | n.s. |
|  | 1 | 3 | 3.0% | 2 | 3.8% |  |
|  | 2 | 4 | 4.0% | 1 | 1.9% |  |
|  | 3 | 3 | 3.0% | 1 | 1.9% |  |
| Infection | 0 | 80 | 79.2% | 45 | 84.9% | n.s. |
|  | 3 | 21 | 20.8% | 8 | 15.1% |  |
| Hearing loss | 0 | 99 | 98.0% | 53 | 100.0% | n.s. |
|  | 1 | 2 | 2.0% | 0 | 0.0% |  |
| Thrombosis | 0 | 98 | 97.0% | 52 | 98.1% | n.s. |
|  | 1 | 1 | 1.0% | 0 | 0.0% |  |
|  | 3 | 1 | 1.0% | 0 | 0.0% |  |
|  | 4 | 1 | 1.0% | 1 | 1.9% |  |
| Hoarseness | 0 | 72 | 71.3% | 45 | 84.9% | n.s. |
|  | 1 | 18 | 17.8% | 5 | 9.4% |  |
|  | 2 | 11 | 10.9% | 3 | 5.7% |  |
| Xerostomia | 0 | 23 | 22.8% | 16 | 30.2% | n.s. |
|  | 1 | 44 | 43.6% | 21 | 39.6% |  |
|  | 2 | 34 | 33.7% | 15 | 28.3% |  |
|  | 3 | 0 | 0.0% | 1 | 1.9% |  |
