# Supplementary figures and images for: Adjuvant chemoradiotherapy in elderly patients with head and neck cancer: a monoinstitutional, two-to-one pair-matching analysis
Source: Strahlenther Onkol. 2022 Jan 17;198(2):159–70. doi: 10.1007/s00066-021-01890-2 (PMC8789714; doi:10.1007/s00066-021-01890-2)

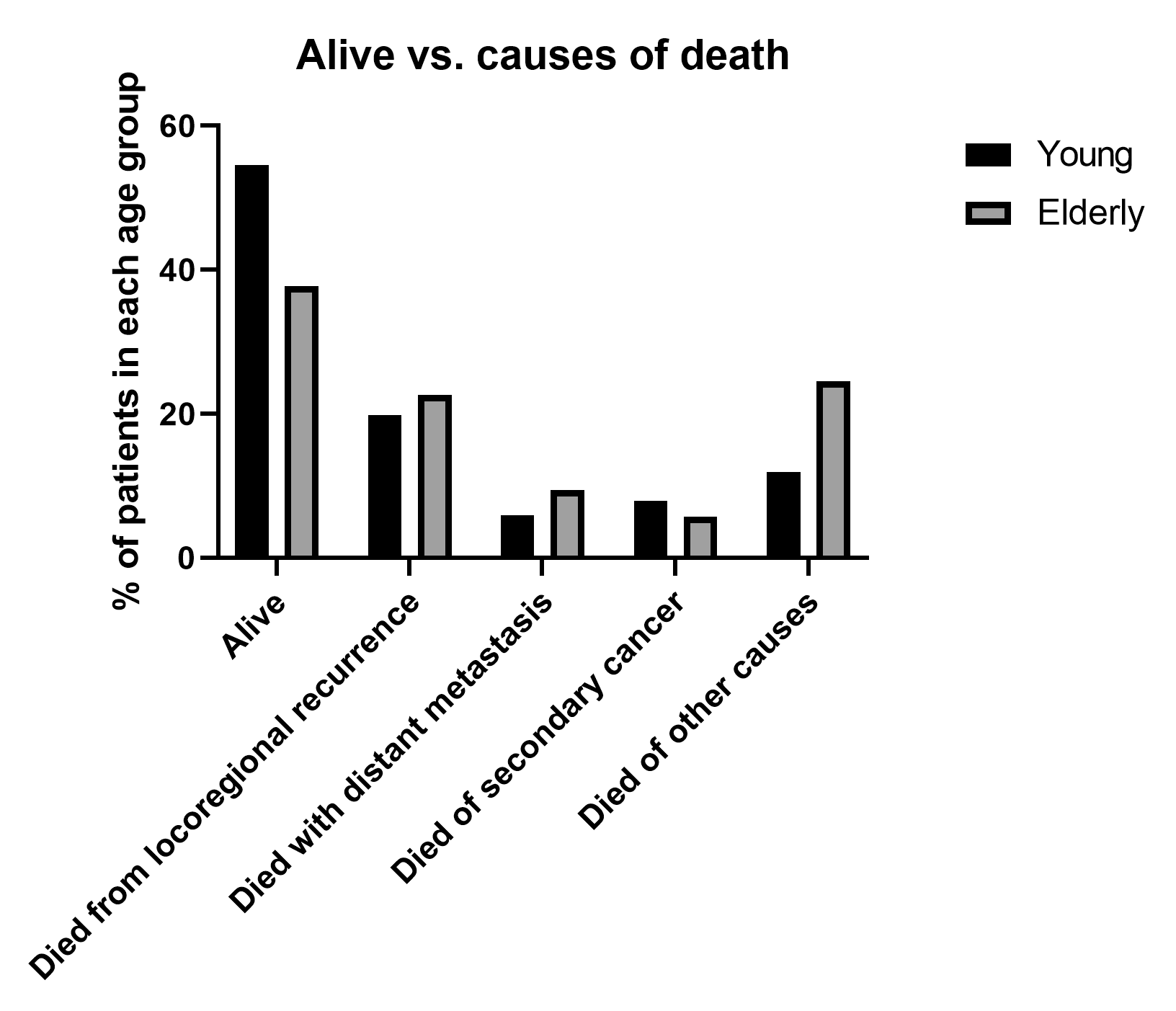

Supplement: Supplementary file 2 — Supplementary Fig. 1. Distributions of survival status and causes of death for the two age groups [file 66_2021_1890_MOESM2_ESM.tif]

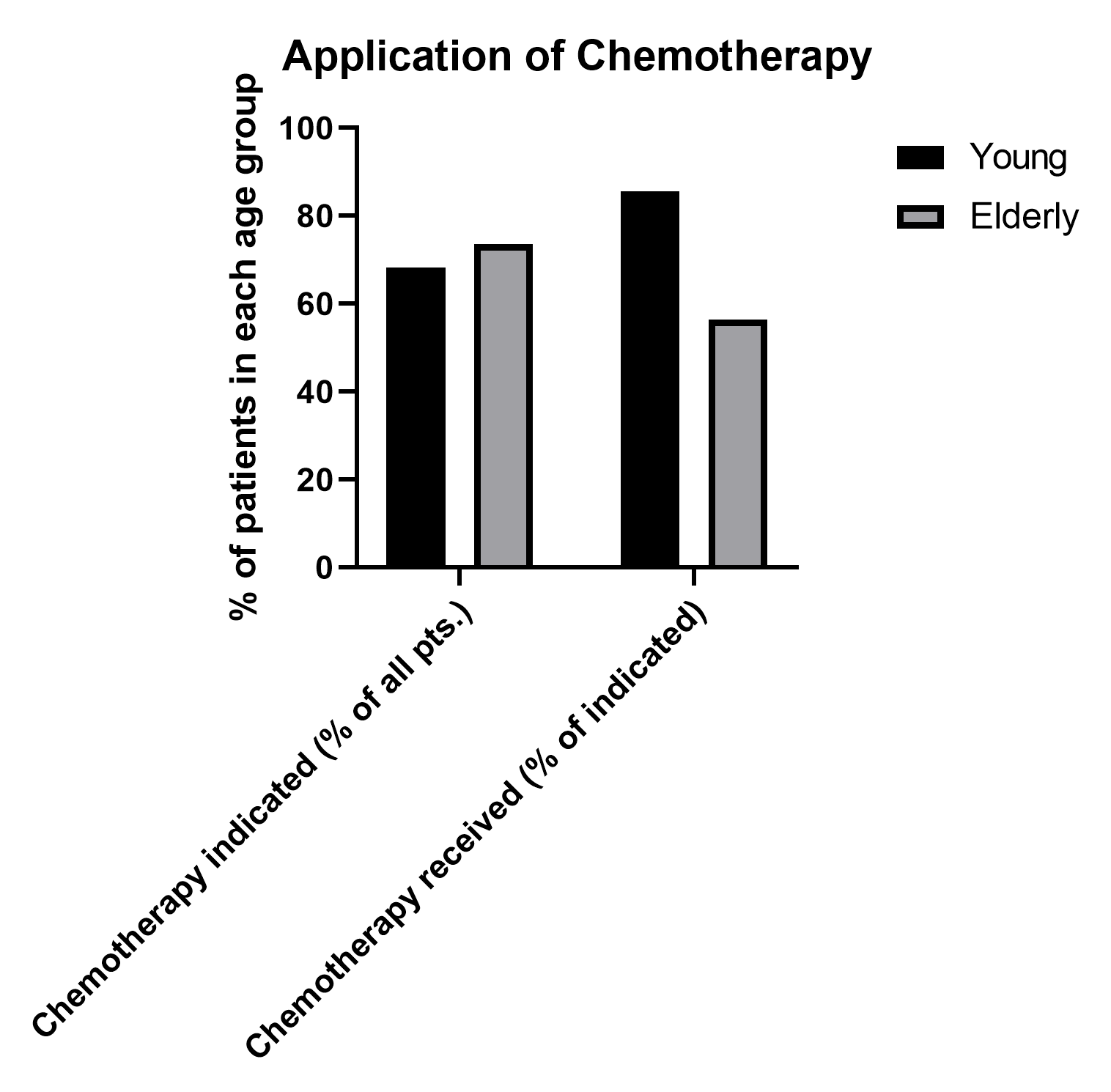

Supplement: Supplementary file 3 — Supplementary Fig. 2. Indications for chemotherapy and the use of chemotherapy in the two age groups [file 66_2021_1890_MOESM3_ESM.tif]
